# Vezatin regulates seizures by controlling AMPAR-mediated synaptic activity

**DOI:** 10.1038/s41419-021-04233-2

**Published:** 2021-10-12

**Authors:** You Wang, Jinxian Yuan, Xinyuan Yu, Xi Liu, Changhong Tan, Yangmei Chen, Tao Xu

**Affiliations:** 1 Department of Neurology, the Second Affiliated Hospital of Chongqing Medical University, 400010 Chongqing, China; 2 Department of Neurology, Chongqing Hospital of Traditional Chinese Medicine, 400021 Chongqing, China

**Keywords:** Cellular neuroscience, Molecular neuroscience

## Abstract

Although many studies have explored the mechanism of epilepsy, it remains unclear and deserves further investigation. Vezatin has been reported to be a synaptic regulatory protein involved in regulating neuronal synaptic transmission (NST). However, the role of vezatin in epilepsy remains unknown. Therefore, the aims of this study are to investigate the underlying roles of vezatin in epilepsy. In this study, vezatin expression was increased in hippocampal tissues from pilocarpine (PILO)-induced epileptic mice and a Mg^2+^-free medium-induced in vitro seizure-like model. Vezatin knockdown suppressed seizure activity in PILO-induced epileptic mice. Mechanistically, vezatin knockdown suppressed AMPAR-mediated synaptic events in epileptic mice and downregulated the surface expression of the AMPAR GluA1 subunit (GluA1). Interestingly, vezatin knockdown decreased the phosphorylation of GluA1 at serine 845 and reduced protein kinase A (PKA) phosphorylation; when PKA phosphorylation was suppressed by H-89 (a selective inhibitor of PKA phosphorylation) in vitro, the effects of vezatin knockdown on reducing the phosphorylation of GluA1 at serine 845 and the surface expression of GluA1 were blocked. Finally, we investigated the pattern of vezatin in brain tissues from patients with temporal lobe epilepsy (TLE), and we found that vezatin expression was also increased in patients with TLE. In summary, the vezatin expression pattern is abnormal in individuals with epilepsy, and vezatin regulates seizure activity by affecting AMPAR-mediated NST and the surface expression of GluA1, which is involved in PKA-mediated phosphorylation of GluA1 at serine 845, indicating that vezatin-mediated regulation of epileptic seizures represents a novel target for epilepsy.

## Introduction

Epilepsy is a heterogeneous disease with a complicated etiology and mechanism [1, 2]. Although many studies have explored the underlying mechanism of epilepsy, the etiology and mechanism of epilepsy remain unclear and require further investigation to potentially aid in the development of new disease-modifying therapies to suppress seizures in patients with epilepsy.

Vezatin, a transmembrane protein expressed in the mammalian brain, was found to be enriched in hippocampal neurons in mice and is associated with anxiety-related behaviors in mice [3, 4]. Previous studies have indicated that vezatin is essential for the formation of cell junctions, which may influence intercellular signal transmission [5, 6]. Vezatin was found to be expressed at high levels in neuronal dendritic spines and regulates synaptic plasticity in vivo and in vitro [3, 4]. Furthermore, altered expression of vezatin affects glutamate receptor-mediated neuronal synaptic transmission (NST) [3]. NST dysfunction plays a vital role in the pathophysiological mechanism of epilepsy [2, 7]. Abnormal NST contributes to an imbalance between neuronal excitation and inhibition, further promoting seizure generation and propagation and even epileptogenesis [2, 7]. Therefore, based on the abovementioned functions of vezatin, we hypothesized that vezatin may play a specific role in epilepsy by influencing NST.

Hence, the aim of this study was to investigate the role of vezatin in regulating seizure activity in a mouse model of pilocarpine (PILO)-induced epilepsy and further explore the molecular mechanism by which vezatin regulates seizure activity.

## Results

### Expression pattern of vezatin in epileptic models in vivo and in vitro

**Fig. 1:**
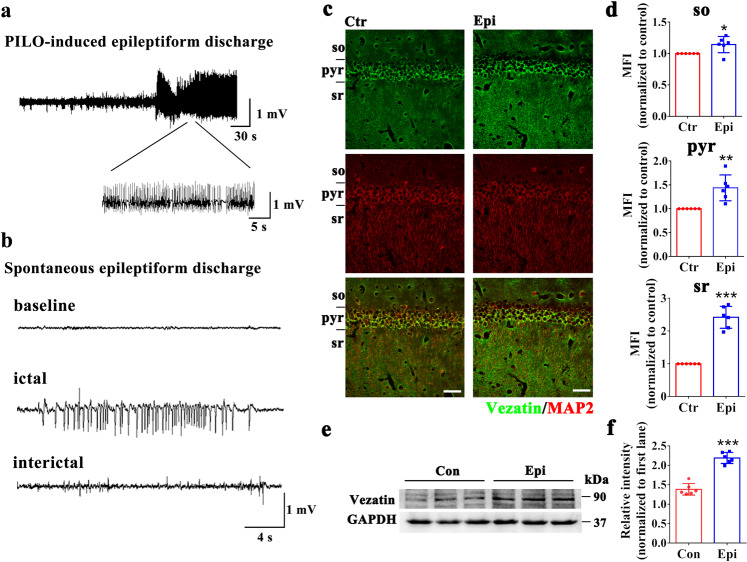
The pattern of vezatin expression in the PILO-induced epilepsy model. **a** Representative image of PILO-induced epileptiform discharges in the acute stage and (**b**) representative image of spontaneous epileptiform discharges in the chronic stage. **c** Representative images of immunofluorescence staining demonstrating that in both the control group and epilepsy group, vezatin (green) was expressed in the hippocampal CA1 region, which was divided into three layers: the stratum oriens (so), pyramidal cell layer (pyr), and stratum radiatum (sr). Vezatin (green) was colocalized with the neuronal and dendritic marker MAP2 (red). **d** Statistical analysis of the MFI of vezatin in the three layers of the CA1 region (*n* = 6 per group; so, *P* = 0.043; pyr, *P* = 0.003; sr, *P* < 0.001). **e** Representative images of western blots showing hippocampal vezatin expression in the control (Con) group and epilepsy (Epi) group and (**f**) the corresponding statistical analysis (*n* = 6 per group, *P* < 0.001). Student’s *t* test; *P < 0.05, **P < 0.01, and ***P < 0.001.

We investigate the expression pattern of vezatin in a mouse model of PILO-induced epilepsy. Hippocampal local-field potentials (LFPs) (Fig. 1a, b) were recorded to confirm the induction of PILO-induced epilepsy in mice. Immunofluorescence staining showed that vezatin (green) expressed in the hippocampal CA1 region in mice colocalized with the neuronal marker microtubule-associated protein 2 (MAP2) (red) (Fig. 1c); the mean fluorescence intensity (MFI) of vezatin was higher in the epilepsy group than in the control group (especially in the stratum radiatum), indicating increased expression of vezatin in the epilepsy model (Fig. 1d). The further western blotting analysis confirmed that the expression of vezatin was increased in the epilepsy group (Fig. 1e, f).

**Fig. 2:**
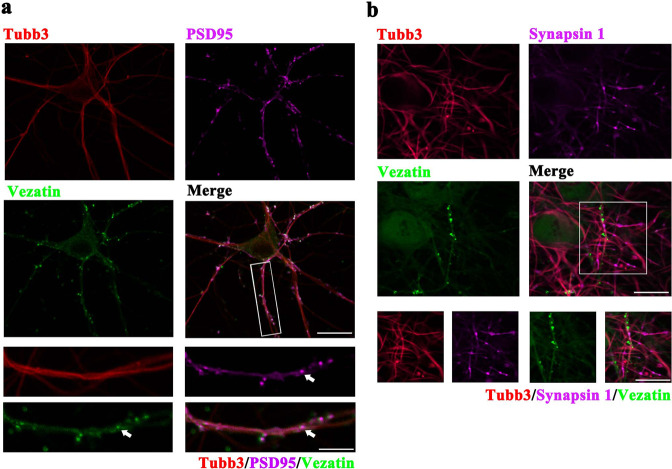
The pattern of vezatin expression in hippocampal neurons in vitro. Representative images of immunofluorescence staining demonstrating that vezatin (green) was localized in neurons positive for the neuronal marker Tubb3 (red) and colocalized with the excitatory postsynaptic marker PSD-95 (purple) (**a**) but not with the excitatory presynaptic marker synapsin 1 (purple) (**b**).

In cultured hippocampal neurons, immunofluorescence staining showed that vezatin (green) was expressed in neurons positive for the neuronal marker beta-Tubulin III (Tubb3) (red) and colocalized with the excitatory postsynaptic marker postsynaptic density protein 95 (PSD-95) (purple) (Fig. 2a) but not with the presynaptic marker synapsin 1 (purple) (Fig. 2b). Immunofluorescence staining indicated a higher fluorescence intensity of vezatin in the Mg^2+^-free medium-induced in vitro seizure-like model than in the control group (Supplementary Fig. S1). Furthermore, western blot analysis also indicated increased expression of vezatin in the in vitro seizure-like model group (Supplementary Fig. S1).

### Lentivirus-mediated knockdown of vezatin in vivo and in vitro

After 2 weeks of lentiviral vectors (LVs) injection into the hippocampal CA1 region of mice, immunofluorescence staining showed the LVs (green) were widely distributed in the hippocampal CA1 region, indicating successful delivery of the LVs to this region (Supplementary Fig. S2). Western blot analysis showed a lower expression level of vezatin in the shRNA group than that in the Scr-seq group at 2 and 4 weeks after LVs injection (Supplementary Fig. S2), indicating that the LVs expressing the shRNA targeting vezatin reduced the expression of vezatin in the mouse hippocampal region.

In primary cultured hippocampal neurons, the LVs successfully infected neurons on day in vitro (DIV) 10 (Supplementary Fig. S3). Western blot analysis revealed a lower expression level of vezatin in the shRNA group than that in the Scr-seq group (Supplementary Fig. S3), indicating that the LVs expressing the shRNA targeting vezatin reduced vezatin expression in cultured neurons.

### The role of vezatin in regulating seizure activity

**Fig. 3:**
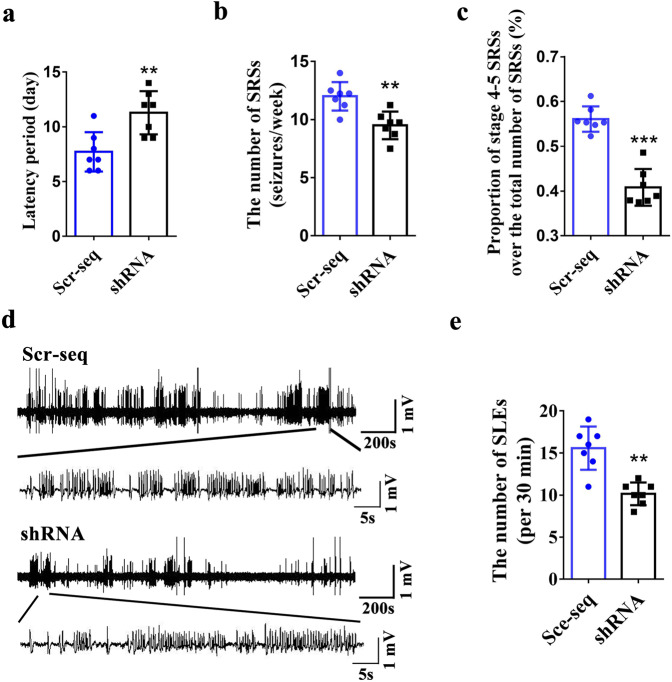
The effect of vezatin knockdown on seizure activity in a mouse model of PILO-induced epilepsy. **a** Statistical analysis of the spontaneous recurrent seizures (SRSs) latency (*P* = 0.004), **b** the number of SRSs (seizures/week) (*P* = 0.002), and **c** the proportion of Racine stage 4–5 SRSs relative to the total number of SRSs (%) (*P* < 0.001). **d** Representative images comparing hippocampal LFP recordings between the Scr-seq group and shRNA group and (**e**) the corresponding statistical analysis of the number of SLEs per 30 min, as identified from hippocampal LFP recordings (*n* = 7 per group, *P* = 0.003). Student’s *t* test; *P < 0.05, **P < 0.01, and ***P < 0.001.

Vezatin knockdown in the hippocampal CA1 region of a mouse model of PILO-induced epilepsy prolonged the SRS latency and reduced the number of SRSs per week and the proportion of stage 4–5 convulsive SRSs (Fig. 3a–c), indicating that vezatin knockdown suppressed seizure activity in epilepsy model. Moreover, hippocampal LFP recordings showed that the number of seizure-like events (SLEs) per 30 min in the shRNA group was lower than that in the Scr-seq group (Fig. 3d, e), indicating that vezatin knockdown inhibited epileptiform discharges in a mouse model of PILO-induced epilepsy. In the in vitro seizure-like model, the frequency of spontaneous epileptiform discharges (SEDs) was lower in the shRNA group than in the Scr-seq group (Supplementary Fig. S4), indicating that vezatin knockdown reduced epileptiform discharges in an in vitro seizure-like model.

### Role of vezatin in the regulation of NST

**Fig. 4:**
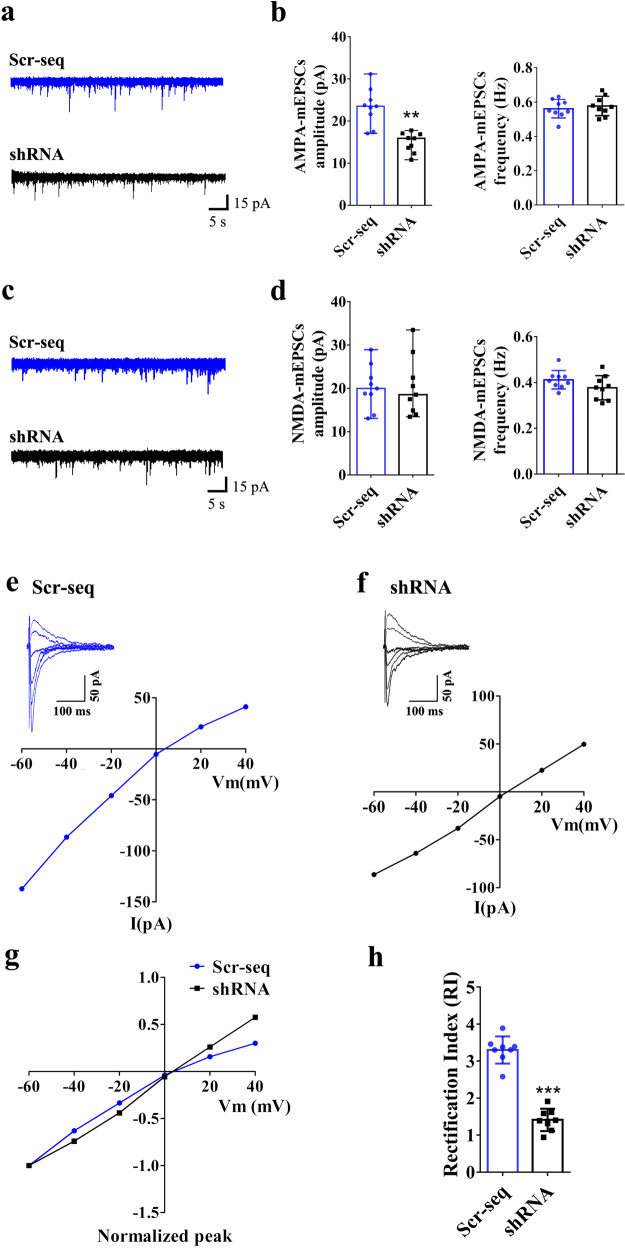
The effect of vezatin knockdown on AMPAR- and NMDAR-mediated synaptic currents in the hippocampal CA1 neurons of epileptic mice. **a** Representative images of AMPAR-mEPSCs in the Scr-seq group and shRNA group and (**b**) the corresponding statistical analysis of the amplitude (presented as medians and ranges; *P* = 0.003; Kruskal–Wallis test; **P < 0.01) and frequency of AMPAR-mEPSCs (*n* = 9 per group). **c** Representative images of NMDAR-mEPSCs in the Scr-seq group and shRNA group and (**d**) the corresponding statistical analysis of the amplitude (presented as medians and ranges; Kruskal–Wallis test) and frequency of NMDAR-mEPSCs (*n* = 9 per group). Current–voltage (I–V) relationships obtained by plotting the amplitudes of AMPAR-eEPSCs at holding potentials ranging from −60 mV to +40 mV in the Scr-seq group (**e**) and in the shRNA group (**f**). The normalized I–V curves of AMPAR-eEPSCs (normalized to the amplitude evoked at −60 mV) (**g**) and statistical analysis of the Scr-seq group and shRNA group (**h**). The RI was calculated from the I–V curves as the ratio of the AMPAR-eEPSC amplitude at −60 mV to the AMPAR-eEPSC amplitude at +40 mV (*n* = 8 per group; *P* < 0.001; Student’s *t* test; ***P < 0.001).

Due to the colocalization of vezatin with the excitatory postsynaptic marker PSD-95 and the role of vezatin in regulating seizures, we postulated that vezatin plays a role in regulating NST in epilepsy and investigated the underlying mechanism. After seizures were recorded, mice were sacrificed to obtain whole-cell patch-clamp recordings. First, we measured glutamatergic NST, including α-amino-3-hydroxy-5-methyl-4-isoxazolepropionic acid receptor (AMPAR)-mediated and N-methyl-d-aspartate receptors (NMDAR)-mediated NST, in hippocampal CA1 neurons of epileptic mice. AMPAR-mediated miniature excitatory postsynaptic current (AMPAR-mEPSC) recordings revealed that vezatin knockdown reduced the amplitude of AMPAR-mEPSCs but did not alter the frequency of AMPAR-mEPSCs (Fig. 4a, b). No significant differences in the amplitude and frequency of NMDAR-mediated mEPSCs (NMDAR-mEPSCs) were observed between the Scr-seq group and shRNA group (Fig. 4c, d). Moreover, we obtained miniature inhibitory postsynaptic current (mIPSC) recordings to investigate the role of vezatin in regulating inhibitory synaptic transmission in hippocampal CA1 neurons. Vezatin knockdown did not alter the amplitude or frequency of mIPSCs (Supplementary Fig. S5).

CP-AMPARs, also called AMPAR GluA2 subunit (GluA2)-lacking AMPARs, play a vital role in determining the strength of AMPAR-mediated NST [8]. Due to the effect of knockdown of vezatin on AMPAR-mEPSCs, we hypothesized that vezatin may influence AMPAR-mediated NST by regulating CP-AMPARs. Thus, we recorded AMPAR-mediated evoked excitatory postsynaptic currents (AMPAR-eEPSCs) and compared the I–V curve of the AMPAR-eEPSCs in hippocampal CA1 neurons from the Scr-seq group and shRNA group. In recordings obtained from the Scr-seq group, the amplitude of AMPAR-eEPSCs evoked at +40 mV was smaller than that recorded at −40 mV (Fig. 4e), indicating that there was an increase in the inward rectification of AMPAR-mediated currents. In recordings obtained from the shRNA group, the amplitude of AMPAR-eEPSCs evoked at +40 mV was equal to that of AMPAR-eEPSCs recorded at -40 mV (Fig. 4f). The normalized I–V curves of AMPAR-eEPSCs also indicated obvious inward rectification at positive holding potentials in the Scr-seq group; in the shRNA group, however, the normalized I–V curves were linear, and no obvious inward rectification was detected at positive holding potentials (Fig. 4g). The RI of hippocampal CA1 neurons in the shRNA group was significantly lower than that of hippocampal CA1 neurons in the Scr-seq group (Fig. 4h). Based on these data, vezatin knockdown in the hippocampal CA1 region suppresses CP-AMPAR activity, indicating that vezatin may regulate AMPAR-mediated NST by influencing GluA2-lacking AMPARs.

### Vezatin modulates the surface expression of AMPAR GluA1 subunit (GluA1) by regulating the phosphorylation levels of GluA1 at serine 845

**Fig. 5:**
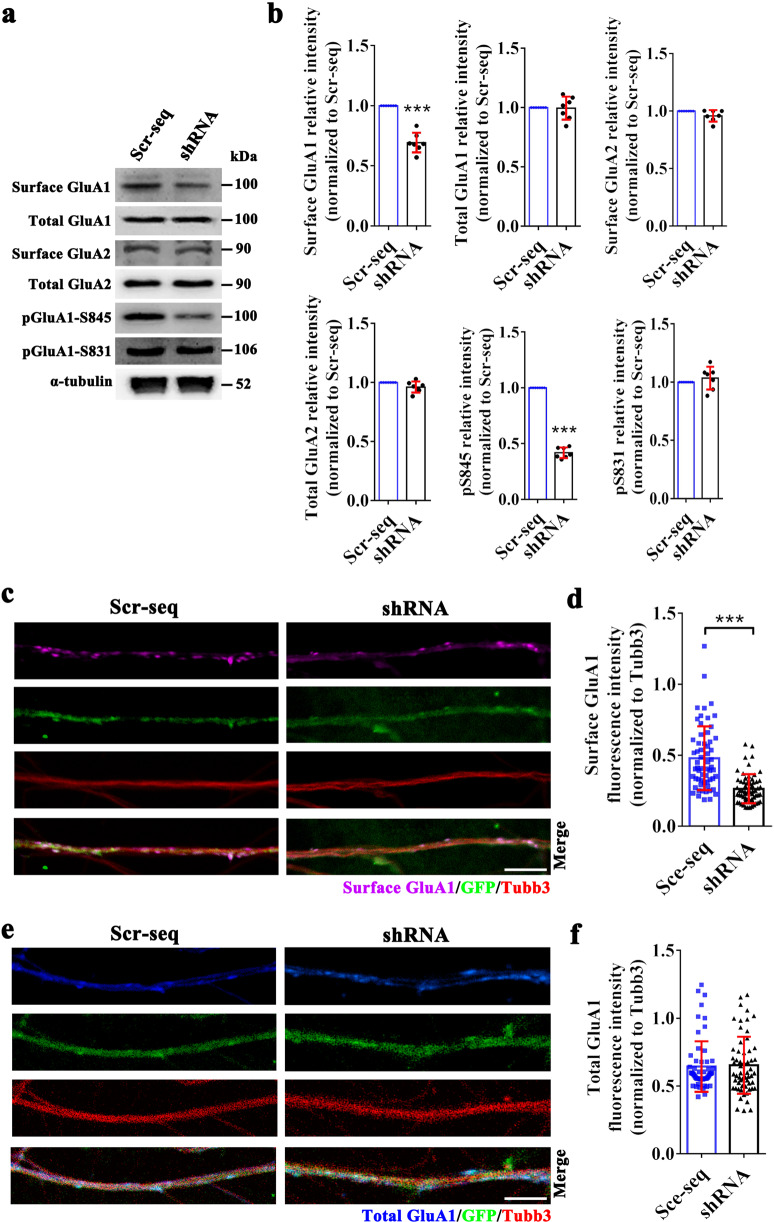
The effect of vezatin knockdown on the expression levels of GluA1, GluA2, pGluA1-S845, and pGluA1-S831. In the PILO-induced epilepsy model: **a** representative images of western blots showing surface GluA1, total GluA1, surface GluA2, total GluA2, pGluA1-S845, and pGluA1-S831 levels in the hippocampus in the Scr-seq group and shRNA group and **b** the corresponding statistical analyses (*n* = 7 per group; surface GluA1, *P* < 0.001; pGluA1-S845, *P* < 0.001). In the Mg^2+^-free solution-induced in vitro seizure-like model, **c** representative images of immunofluorescence staining showing that surface GluA1 (purple) was localized in Tubb3 (red)-positive and LV-infected neuronal dendrites (green) and (**d**) the corresponding statistical analysis of the fluorescence intensity of surface GluA1 in the Scr-seq group and shRNA group (*P* < 0.001). **e** Representative images of immunofluorescence staining showing that total GluA1 (blue) was localized in Tubb3 (red)-positive and LV-infected (green) neuronal dendrites and (**f**) the corresponding statistical analysis of the fluorescence intensity of total GluA1 expression in the Scr-seq group and shRNA group (*n* = 5 independent hippocampal neuron cultures from ten mice per group). Student’s *t* test; *P < 0.05, **P < 0.01, and ***P < 0.001.

We further assessed the effect of vezatin knockdown on the expression levels of two key AMPAR subunits, GluA1 and GluA2. Vezatin knockdown reduced the surface expression of GluA1 but not the expression level of total GluA1 in the hippocampi of epileptic mice (Fig. 5a, b). Vezatin knockdown did not alter the surface expression levels of GluA2 or the total expression of GluA2 in the hippocampi of epileptic mice (Fig. 5a, b). Moreover, in the in vitro seizure-like model, immunofluorescence staining indicated that vezatin knockdown decreased the surface expression level of GluA1 (Fig. 5c, d) but did not alter the total GluA1-expression level (Fig. 5e, f). These data indicate a role for vezatin in regulating the surface expression of GluA1 in vivo and in vitro. The major phosphorylation sites of GluA1 are serine 845 and serine 831; phosphorylation of these sites, phospho-GluA1 serine 845 (pGluA1-S845) and phospho-GluA1 serine 831 (pGluA1-S831), regulates the targeting of GluA1 to or its retention at the cell surface, further affecting the accumulation of surface GluA1 [9, 10]. Thus, we further evaluated the effect of vezatin knockdown on levels of pGluA1-S845 and pGluA1-S831 in mice. Vezatin knockdown reduced the level of pGluA1-S845 but not pGluA1-S831 (Fig. 5a, b), suggesting that vezatin regulates the surface expression of GluA1 by influencing the levels of pGluA1-S845.

### Vezatin modulates the level of pGluA1-S845 by influencing protein kinase A (PKA) activity

**Fig. 6:**
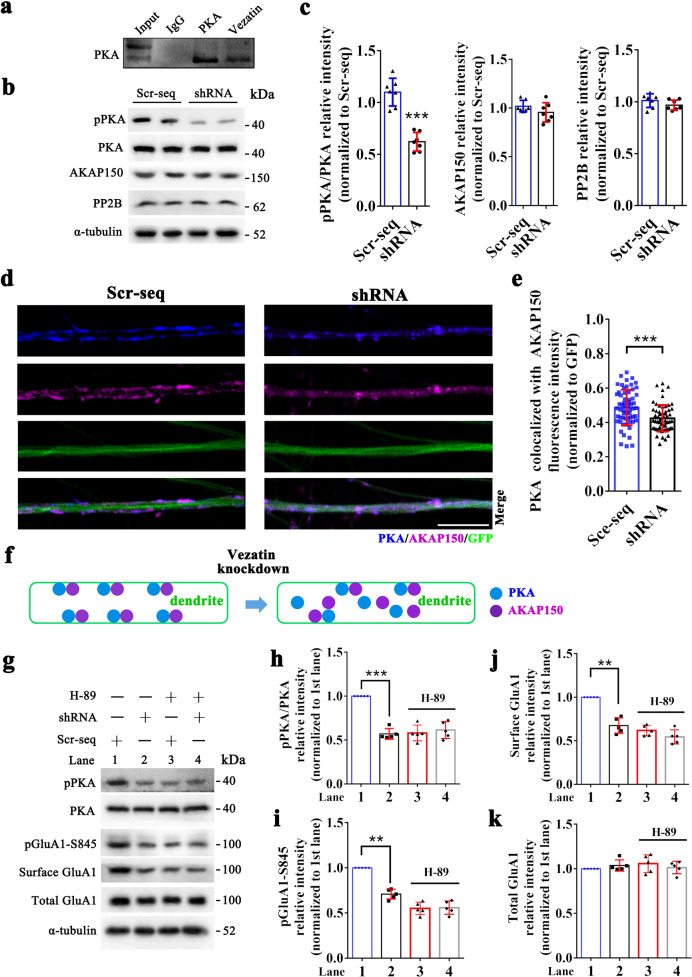
PKA signaling mediates the regulatory effect of vezatin on the phosphorylation of GluA1 at serine 845. **a** Representative coimmunoprecipitation image showing the interaction between vezatin and PKA in the hippocampi of mice. In the PILO-induced epilepsy model, **b** representative images of western blots showing pPKA levels, which was calculated as the pPKA to total PKA ratio, and the expression of AKAP150 and PP2B in the hippocampus of the Scr-seq group and shRNA group and **c** the corresponding statistical analyses (*n* = 7 per group; pPKA/PKA, *P* < 0.001; Student’s *t* test; ***P < 0.001). In the Mg^2+^-free medium-induced in vitro seizure-like model, **d** representative images of immunofluorescence staining comparing the distribution and colocalization (blue–purple) of PKA (blue) and AKAP150 (purple) in LV-infected neuronal dendrites (green) between the Scr-seq group and shRNA group and (**e**) the corresponding statistical analysis of the fluorescence intensity of the colocalization of PKA and AKAP150 between the Scr-seq group and shRNA group (*P* < 0.001; Student’s *t* test; ***P < 0.001) (*n* = 5 independent hippocampal neuron cultures from ten mice per group). **f** The corresponding schematic of the effect of vezatin knockdown on the distribution and colocalization of PKA and AKAP150 is also shown. In the Mg^2+^-free medium-induced in vitro seizure-like model, **g** representative images of western blots show that H-89 blocks the effect of vezatin knockdown on the level of pGluA1-S845 and the surface expression of GluA1, along with the corresponding statistical analyses of pPKA/PKA (*P* < 0.001) (**h**), pGluA1-S845 (*P* = 0.001) (**i**), surface GluA1 (*P* = 0.003) (**j**), and total GluA1 expression (**k**) (*n* = 5 independent hippocampal neuron cultures from ten mice per group). Two-way ANOVA followed by the Bonferroni post hoc test; *P < 0.05, **P < 0.01, and ***P < 0.001.

The level of pGluA1-S845 is mainly determined by PKA- and calcium/calmodulin-dependent kinase II-α (CaMKII-α)-mediated phosphorylation of GluA1 at serine 845 and by protein phosphatase (PP) 2B-, PP2A-, and PP1-mediated dephosphorylation of GluA1 at serine 845 [8, 11]. We postulated that vezatin plays a role in regulating the abovementioned signaling mechanisms, further affecting the level of pGluA1-S845. First, the coimmunoprecipitation analysis indicated that vezatin interacts with PKA (Fig. 6a) and CaMKII-α (Supplementary Fig. S6) but not PP2B, PP2A, or PP1 in the mouse hippocampus (Supplementary Fig. S6). Vezatin knockdown in the hippocampi of epileptic mice reduced the level of phospho-PKA (pPKA) (Fig. 6b, c) but did not alter the level of CaMKII-α phosphorylation (Supplementary Fig. S6) or the expression levels of PP2B (Fig. 6b, c), PP2A and PP1 (Supplementary Fig. S6). Based on these data, we speculated that vezatin plays a role in regulating the activation of PKA signaling, further affecting the level of pGluA1-S845. AKAP150 is known to anchor PKA when it targets pGluA1-S845 [8, 12], which may be involved in the regulation of PKA signaling by vezatin. Thus, we also evaluated the interaction between vezatin and AKAP150. The coimmunoprecipitation analysis indicated that vezatin interacts with AKAP150 (Supplementary Fig. S6); however, vezatin knockdown did not alter the expression of AKAP150 in the hippocampi of epileptic mice (Fig. 6b, c).

Next, we explored the underlying mechanism by which vezatin regulates PKA signaling in a vitro seizure-like model. Immunofluorescence staining showed that vezatin (purple) colocalized with PKA (blue) (Supplementary Fig. S7). Interestingly, vezatin knockdown reduced the colocalization of PKA and AKAP150 (Fig. 6d, e); moreover, vezatin knockdown promoted the internalization of PKA (blue) and AKAP150 (purple) in LV-infected neuronal dendrites (green) (Fig. 6d, f). These data suggest that vezatin knockdown inhibits the activation of PKA signaling and promotes the internalization of PKA and AKAP150 in epilepsy models.

To further confirm whether PKA mediates the effect of vezatin on the level of pGluA1-S845, H-89 (a selective inhibitor of PKA phosphorylation) was applied to inhibit the phosphorylation of PKA in cultured neurons in vitro. Western blot analysis showed that compared with the Scr-seq group that was not treated with H-89, the H-89-treated Scr-seq group exhibited a decreased level of pPKA, indicating that H-89 successfully suppressed the phosphorylation of PKA (Fig. 6g, h). No significant difference in the level of pPKA was observed between the Scr-seq group and shRNA group after treatment with H-89 (Fig. 6h), indicating that H-89 blocked the effect of vezatin knockdown on the phosphorylation of PKA. Furthermore, in the groups that was not treated with H-89, vezatin knockdown reduced the level of pGluA1-S845 (Fig. 6i) and the surface expression of GluA1 (Fig. 6j); however, upon treatment with H-89, no significant differences in the level of pGluA1-S845 (Fig. 6i) and the surface expression of GluA1 (Fig. 6j) were observed between the Scr-seq group and shRNA group. No significant difference in the total expression of GluA1 was observed among all the groups (Fig. 6k). These data indicate that H-89 can block the downregulatory effect of vezatin knockdown on the expression of pGluA1-S845 and the surface expression of GluA1.

### Expression pattern of vezatin in subjects with epilepsy

**Fig. 7:**
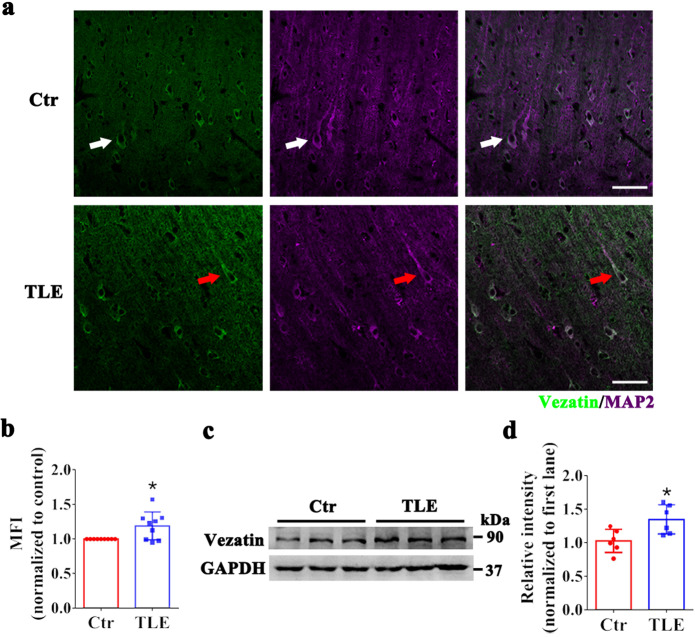
The pattern of vezatin expression in subjects with epilepsy. **a** Representative images of immunofluorescence staining showing that in both the control group (white arrow) and TLE group (red arrow), vezatin (green) colocalized with the neuronal marker MAP2 (purple), and **b** the corresponding statistical analyses of the MFI of vezatin (*n* = 9 per group, *P* = 0.023). **c** Representative images of western blots showing vezatin expression in the control group and TLE group and (**d**) the corresponding statistical analysis (*n* = 6 per group, *P* = 0.017). Student’s *t* test; *P < 0.05, **P < 0.01, and ***P < 0.001.

Vezatin expression in patients with temporal lobe epilepsy (TLE) was measured to further investigate the expression pattern of vezatin in epilepsy. Eleven controls with traumatic brain injury (TBI), i.e., six females and five males with a mean age of 29.00 ± 3.70 years (mean ± standard error), comprised the control group (Supplementary Table S1). Eleven patients with TLE, i.e., six females and five males with a mean age of 23.27 ± 2.03 years (mean ± standard error), were included in the TLE group (Supplementary Table S2). There was no significant difference in age (*P* = 0.126) or sex (*P* = 1.000) between the two groups. Immunofluorescence staining showed that vezatin (green) was colocalized with the neuronal marker MAP2 (purple) in both the TLE group and the control group (Fig. 7a). Moreover, the fluorescence intensity of vezatin in the TLE group was higher than that in the control group (Fig. 7b). A subsequent western blot analysis suggested increased vezatin levels in the TLE group (Fig. 7c, d).

## Discussion

This study investigated the roles of vezatin in epilepsy. First, our study showed a significant increase in vezatin expression in the hippocampal tissues from epileptic mice and in an in vitro seizure-like model. Subsequent behavioral tests revealed that vezatin knockdown suppressed seizure activity in epileptic mice. We further explored the underlying mechanism by which vezatin regulates seizure activity. Subsequent electrophysiological studies revealed that vezatin knockdown suppressed CP-AMPAR-mediated synaptic events and inhibited the surface expression of GluA1 in epileptic mice, indicating that vezatin may regulate AMPAR-mediated synaptic events by affecting the surface expression of GluA1. The accumulation of surface GluA1 is largely determined by the level of pGluA1-S845 [8]. Interestingly, vezatin knockdown decreased the level of pGluA1-S845; moreover, vezatin knockdown reduced the phosphorylation of PKA, an important regulator that promotes phosphorylation of GluA1 at serine 845 [8, 11]. When the phosphorylation of PKA was suppressed by H-89, the effects of vezatin knockdown on the phosphorylation of GluA1 at serine 845 and the surface expression of GluA1 were blocked, indicating that vezatin could modulate the level of pGluA1-S845, further regulating the surface expression of GluA1, which requires the activation of PKA signaling. Finally, we found that vezatin expression was increased in brain tissues from patients with TLE, which suggests a possible relationship between vezatin and epilepsy in humans.

Vezatin is a synaptic regulatory protein that can regulate NST [3, 4]. In this study, vezatin expression was increased in the hippocampal CA1 region in epileptic mice, especially in the stratum radiatum. The hippocampal CA1 region contains many pyramidal neurons, which project dendrites and axons that form synapses with excitatory and inhibitory synaptic inputs mainly located in the stratum radiatum of the CA1 region [2, 13]. Thus, the increased vezatin expression in the hippocampal CA1 region in epileptic mice revealed by our immunofluorescence analyses suggests a possible function for vezatin in regulating NST in epilepsy. Moreover, in vitro immunofluorescence staining indicated that vezatin was localized in the postsynaptic component of excitatory synapses, further suggesting that vezatin regulates seizure activity by affecting NST. Using the whole-cell patch-clamp technique, we determined that vezatin knockdown could decrease the amplitude of AMPAR-mEPSCs in epileptic mice, indicating that vezatin regulates seizure activity by affecting AMPAR-mediated NST. As key glutamate-gated ion channels located on the postsynaptic membrane, AMPARs mediate the majority of fast excitatory NST in the brain, which plays a vital role in the development of epilepsy [8]. CP-AMPARs, the majority of which are likely GluA1 homomers, are widely expressed in synapses during the early development of the brain [8, 11]; with the development of the brain, CP-AMPARs are progressively lost in the majority of synapses on excitatory pyramidal neurons in the mature brain [8, 11]. In the seizure model, however, CP-AMPAR-mediated synaptic events were significantly enhanced [14]. Thus, we postulated that vezatin plays a role in regulating CP-AMPARs that underlies epilepsy. Next, we found that vezatin knockdown could suppress CP-AMPAR-mediated synaptic events in the hippocampi of epileptic mice. Moreover, vezatin knockdown reduced the surface expression of GluA1 in vivo and in vitro. These data indicate that vezatin can affect AMPAR-mediated NST by regulating the surface expression of GluA1 in epilepsy.

**Fig. 8:**
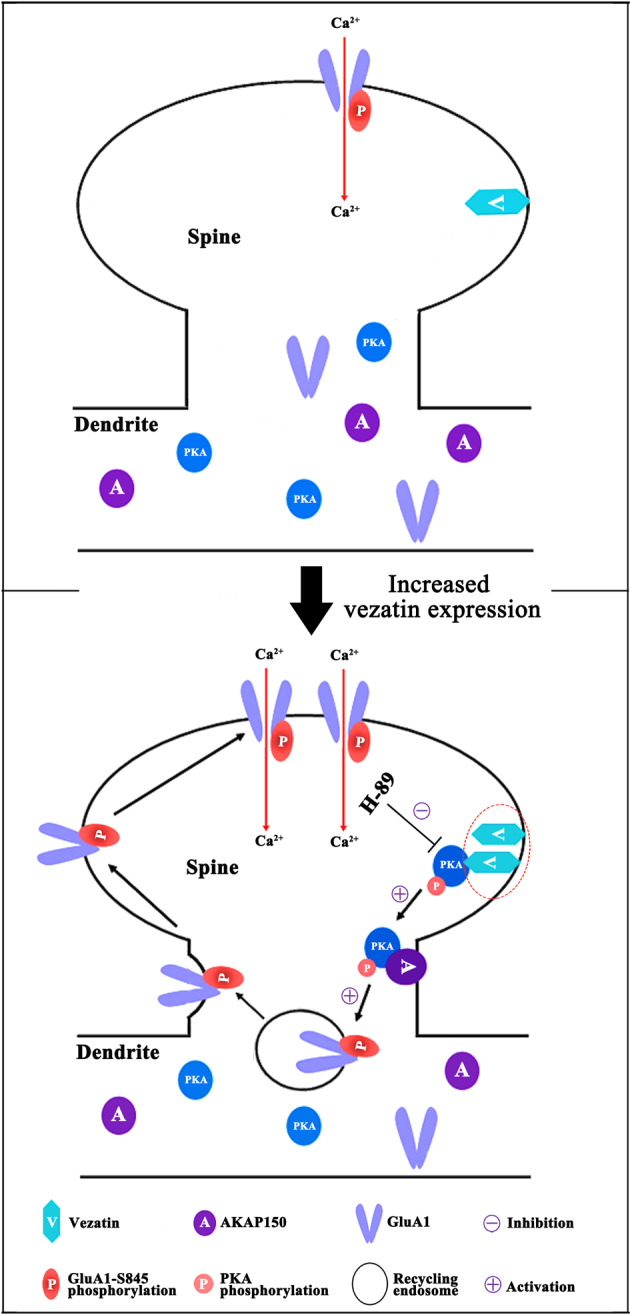
Schematic of the mechanism by which vezatin regulates seizure activity. Increased vezatin expression promotes PKA activation (which can be blocked by H-89), facilitating the targeting of pGluA1-S845 by PKA and AKAP150, further increasing the phosphorylation of GluA1 at serine 845 and promoting the targeting of AMPARs to the neuronal surface and then to postsynaptic elements, ultimately enhancing AMPAR-mediated synaptic transmission and promoting seizure activity in individuals with epilepsy.

Serine 845 is the major phosphorylation site of AMPARs, and phosphorylation at serine 845 largely affects the targeting of AMPARs to or retention of AMPARs at the cell surface [8, 11]. PKA and CaMKII promote the phosphorylation of GluA1 at serine 845, further facilitating the targeting of AMPARs to the cell surface and enhancing AMPAR-mediated NST [8, 11, 12]. In this study, vezatin interacted with PKA and CaMKII-α by coimmunoprecipitation. Furthermore, vezatin knockdown inhibited the phosphorylation of PKA but had no effect on the phosphorylation of CaMKII-α in epileptic mice. These data indicate that PKA may mediate the role of vezatin in regulating the level of pGluA1-S845. The level of pGluA1-S845 is also affected by the expression of the proteins that dephosphorylated GluA1, including PP2B (which plays a major role), PP2A, and PP1 [11, 15]. Coimmunoprecipitation indicated negative interactions between vezatin and the three dephosphorylating proteins; accordingly, vezatin knockdown exerted no effect on the expression levels of these proteins. AKAP150 is known to anchor PKA when it targets pGluA1-S845[12]; dysfunction of AKAP150 might affect the phosphorylation of GluA1 at serine 845 by PKA [12, 16]. Thus, we postulated that there may be a relationship between vezatin and AKAP150. Coimmunoprecipitation revealed an interaction between vezatin and AKAP150; however, vezatin knockdown did not alter the expression of AKAP150 in epileptic mice. Interestingly, subsequent immunofluorescence analyses in the in vitro model indicated that vezatin knockdown decreased the colocalization of PKA with AKAP150, suggesting that vezatin knockdown may inhibit the binding of PKA to AKAP150. Moreover, immunofluorescence staining suggested that vezatin knockdown seemed to promote the internalization of both PKA and AKAP150 in neuronal dendrites in the in vitro seizure-like model, possibly influencing the effect of PKA and AKAP150 on targeting of GluA1 to the cell surface and subsequently affecting AMPAR-mediated synaptic activity. H-89 was used to inhibit the phosphorylation of PKA in the in vitro seizure-like model and to further assess the mediating effect of PKA on the regulation of pGluA1-S845 by vezatin. When H-89 was administered, the downregulatory effects of vezatin knockdown on the expression of pGluA1-S845 and the surface expression of GluA1 were inhibited, indicating that H-89 blocked the effect of vezatin knockdown on the phosphorylation of GluA1 at serine 845, further inhibiting the effect of vezatin knockdown on the surface expression of GluA1. Based on these data, increased vezatin expression promotes PKA activation, further increasing the phosphorylation of GluA1 at serine 845, facilitating the targeting of AMPARs to the cell surface, and ultimately enhancing AMPAR-mediated NST and promoting seizure activity in epilepsy (Fig. 8).

A limitation of this study should be considered when interpreting the results. In the present study, cultured hippocampal neurons were exposed to Mg^2+^-free medium to mimic seizure-like activity in vitro and to explore the underlying mechanism of vezatin in epilepsy. Although this in vitro model has been routinely used to investigate the underlying molecular mechanisms of epilepsy, the cultured hippocampal neurons in vitro did not form anatomical connections or exhibit brain functions and did not display the clinical seizures observed in humans with epilepsy [17]. The PILO-induced mouse model mimicking acquired epilepsy also does not completely represent the complex epileptic condition in humans with epilepsy [18]. Thus, our findings regarding the function and the molecular mechanism of vezatin in regulating seizure activity did not completely reflect the molecular mechanism of vezatin underlying the clinical epileptic condition. The function and the molecular mechanism of vezatin in human epilepsy remain undetermined.

In summary, we demonstrated that the expression pattern of vezatin is abnormal in epilepsy and that vezatin regulates seizure activity by affecting AMPAR-mediated NST and the surface expression of GluA1, which is involved in PKA-mediated phosphorylation of GluA1 at serine 845. These findings may provide a novel target for seizure control.

## Materials and methods

### Animals

All animal experiments performed in this study were approved by the Committee on Animal Research of Chongqing Medical University, Chongqing, China. Healthy adult male C57BL/6 mice (8–10 weeks old, and 20–25 g) were obtained from the Experimental Animal Center of Chongqing Medical University. All mice were raised in a temperature-controlled room (24–26 °C) on a 12 h light/dark cycle and provided free access to food and water.

### Mouse model of PILO-induced epilepsy and behavioral recordings

Epilepsy was induced by PILO according to a method described in a previously published study [18]. PILO-induced seizures were classified according to Racine’s standard criteria (stages 1–5) [19]. Before PILO administration, the mice were intraperitoneally (i.p.) injected with lithium chloride (LiCl, 127 mg/kg, Sigma-Aldrich, USA) for 20 h. Atropine methyl nitrate (1 mg/kg) was i.p. injected 30 min before PILO administration to reduce the peripheral cholinomimetic effects of PILO. To induce status epilepticus (SE), PILO (50 mg/kg, Sigma-Aldrich, USA) was i.p. administered. Only mice that had persistent convulsive SE (stage 4 or 5) were considered for further study. Mice exhibiting convulsive SE for 1 h were i.p. injected with 10 mg/kg diazepam to terminate SE. After the acute SE stage, the SRSs of mice were monitored continuously with a video recording system for 30 consecutive days; SRSs were also classified according to Racine’s criteria [19]. Only mice that exhibited SRSs were included in the epilepsy group. Hippocampal LFPs were also recorded to confirm the induction of PILO-induced epilepsy in mice. Mice in the control group were i.p. administered the same volume of 0.9% saline.

In addition to SE and SRSs, hippocampal LFPs were monitored to confirm the establishment of PILO-induced epilepsy in mice. The procedure for recording LFPs was described previously [20]. Briefly, mice were deeply anesthetized by i.p. injected sodium pentobarbital (50 mg/kg) and mounted on a stereotaxic apparatus (RWD Life Science, China). Electrodes were implanted in the dorsal hippocampal region (anterior–posterior, 2.0 mm; medial–lateral, 1.5 mm; dorsal–ventral, 1.5 mm), and electrodes connected to a signal generator were fixed to the skull with dental acrylic cement. The MAP data acquisition system (Plexon, USA) was used to monitor and record LFPs. LFPs were further analyzed using Neuroexplorer software (Nex Technologies, USA). PILO-induced epileptiform discharges were recorded in the acute SE stage (Fig. 1a). Moreover, we recorded SEDs in vivo, named SLEs, after the acute induced SE stage (Fig. 1b); SLEs were defined as spontaneous paroxysmal polyspike discharges lasting for more than 5 s with a high amplitude (>2 times the baseline value) and a frequency >5 Hz [21, 22].

### LV construction and stereotaxic injection

An LV expressing a shRNA targeting vezatin was constructed to knock down vezatin expression in the hippocampal region. The LV expressed a transgene-encoding green fluorescent protein (GFP). The LV expressing the vezatin-targeting shRNA (AGCCAACTTTCAAGCCGCAAGGCTA) was used to knock down the expression of vezatin in the shRNA group, and an LV expressing a scramble sequence (TTCTCCGAACGTGTCACGTAA) was used as a negative control in the Scr-seq group. All LVs were manufactured by Hanbio Biotechnology (Shanghai, China). The titer of these LVs was 5 × 10^8^ TU/ml.

The procedures for performing i.p. stereotaxic injection have been described previously [20, 23]. Briefly, the mice were deeply anesthetized by an i.p. injection of sodium pentobarbital (50 mg/kg) and mounted on a stereotaxic apparatus. The lentivirus (2 μl) was stereotactically injected into the hippocampal CA1 region (anterior–posterior, 2.0 mm; medial–lateral, 1.3 mm; dorsal–ventral, 1.4 mm) at a speed of 0.4 μl/min with a 5-μl syringe. The syringe was maintained in situ for an additional 5 min and then withdrawn slowly to prevent reflux.

To determine the effects of vezatin knockdown on seizure activity in the PILO-induced epilepsy model, mice were i.p. injected with PILO 2 weeks after hippocampal injection of LV. SRSs were monitored continuously with a video-monitoring system (24 h/day) for 30 consecutive days. We also monitored and recorded hippocampal LFPs. The latency period, frequency, and Racine stage of SRSs and number of SLEs were determined and analyzed independently by two researchers. The SRS latency was defined as the interval between PILO injection and the onset of the first SRS.

### Primary neuron culture and LV transfection

Hippocampal tissues were dissected from postnatal (P0-1) C57BL/6 mice. Hippocampal explants were digested with trypsin and then mechanically triturated. Next, a diluted cell suspension was plated in poly-l-lysine-coated dishes, and the neurons were incubated in a cell culture incubator at 37 °C for 4 h. For hours after plating, the medium was replaced with neurobasal medium supplemented with 2% B27 (Gibco, USA), 1% antibiotics, and 0.5 mM l-glutamine in a humidified incubator at 37 °C [24]. Then, ~1/2 of the culture medium was changed every 3 days.

Cultured hippocampal neurons that display SEDs have been well characterized as a useful in vitro model of seizures [17, 24]. We generated this in vitro seizure model by replacing the culture medium with a Mg^2+^-free extracellular solution (in mM) (145 NaCl, 10 HEPES, 2.5 KCl, 2 CaCl_2_, 10 glucose, 0.002 glycine; pH 7.3; osmolarity adjusted to 320 mOsm) for 3 h on DIV 14. In the control group, the culture medium was replaced with extracellular solution containing 1 mM MgCl_2_. Then, in both groups, the extracellular solution was replaced with fresh culture media. After 24 h, the SEDs of cultured neurons were recorded using the whole-cell patch-clamp technique to confirm the establishment of the in vitro seizure-like model (Supplementary Fig. S1). An SED was defined as a spontaneous high-frequency burst of action potentials (APs) (≥4 APs) [25, 26].

To knockdown the expression of vezatin in vitro, cultured neurons in the shRNA group were transfected with an LV expressing shRNA targeting vezatin on DIV 3. Neurons in the Scr-seq group were transfected with an LV expressing a scramble sequence as a negative control. To determine the effects of vezatin knockdown on SEDs in vitro, transfected neurons were exposed to Mg^2+^-free extracellular solution on DIV14 [24]. After 24 h, the SEDs were recorded using the whole-cell patch-clamp technique.

Cultured neurons were incubated with medium containing 30 μM H-89 (MedChemExpress) for 2 h to inhibit the phosphorylation of PKA in vitro [27] and then with Mg^2+^-free extracellular solution. Then, in both groups, the extracellular solution was replaced with fresh culture media. After 24 h, neurons were harvested for western blot analysis.

### Subjects with epilepsy and controls

The human study complied with the Declaration of Helsinki and the ethical principles of the National Institutes of Health and was approved by the Committee on Human Research of the Second Affiliated Hospital of Chongqing Medical University. All human brain tissue samples were obtained as described in our previous studies [20, 28]. Brain tissue samples were obtained from 11 patients undergoing surgery for medically refractory TLE and 11 controls undergoing surgery for TBI at the Xinqiao Hospital of the Third Military University and the First Affiliated Hospital of Chongqing Medical University, China. Informed consent for the use of brain tissues in this study was obtained from the patients or their lineal relatives. Refractory TLE was diagnosed according to criteria proposed by the International League Against Epilepsy [29]. All patients with refractory TLE had typical symptoms and electroencephalogram (EEG) features, and their seizures were refractory to combination therapy with the maximal tolerable doses of at least three antiepileptic drugs (AEDs) for more than 2 years. The patients with refractory TLE underwent detailed medical evaluations, including assessment of epilepsy manifestations, a neurological examination, neuroimaging (e.g., positron emission tomography-computed tomography or high-resolution magnetic resonance imaging), and electrophysiological examination (24 h video EEG), prior to surgery. The epileptic focus was also localized during surgery via intraoperative EEG. Brain tissue samples were obtained from control group patients, i.e., patients who required craniocerebral surgery for increased intracranial pressure resulting from severe TBI and who had no history of epilepsy, seizures, or exposure to AEDs. The clinical data of these subjects are provided in Supplementary Tables S1 and S2.

### Immunofluorescence staining

The immunofluorescence procedures were described previously [20, 28]. The mice were rapidly euthanized, and mouse brain tissues were fixed with 4% paraformaldehyde for 24 h; the human brain tissues were also fixed with 4% paraformaldehyde for 24 h. The mouse brain tissues and human brain tissues were sequentially incubated in 20% and 30% graded sucrose solutions for 24 h and sliced into 15-μm frozen sections using a freezing microtome. Then, the sections were collected on glass slides. The frozen sections were air-dried at room temperature for 10 min and immersed in acetone for 20 min. Next, the sections were washed with phosphate-buffered saline (PBS) and permeabilized with 0.4% Triton X-100. After that, the sections were blocked with 4% goat serum for 120 min. For immunofluorescence analysis of cultured neurons, the neurons were plated and then allowed to adhere to coverslips; neurons on coverslips were fixed with 4% paraformaldehyde for 10 min and then permeabilized with 0.4% Triton X-100 and blocked with 4% goat serum. The sections and cultured neurons were incubated with primary antibodies overnight at 4 °C. The following primary antibodies were used: a mouse vezatin antibody (Santa Cruz Biotechnology; 1:100), rabbit MAP2 antibody (Proteintech; 1:200), guinea pig Tubb3 antibody (Synaptic Systems; 1:200), mouse Tubb3 antibody (Proteintech; 1:400), rabbit PSD-95 antibody (Abcam; 1:200), rabbit Synapsin 1 antibody (Novus; 1:200), rabbit GluA1 antibody (Abcam; 1:200), rabbit PKA antibody (Cell Signaling Technology; 1:100), and mouse AKAP150 antibody (Santa Cruz Biotechnology; 1:100). The following secondary antibodies were used: fluorescein isothiocyanate (FITC)-labeled goat anti-mouse IgG (Proteintech; 1:100), Alexa Fluor 555-labeled goat anti-rabbit IgG (Beyotime; 1:500), Alexa Fluor 647-labeled goat anti-rabbit IgG (Beyotime; 1:200), Alexa Fluor 555-labeled goat anti-guinea pig IgG (Abcam; 1:500), Alexa Fluor 647-labeled goat anti-mouse IgG (Beyotime; 1:200), and AMCA-labeled goat anti-rabbit IgG (Proteintech; 1:200). Next, the sections and neurons on coverslips were extensively washed with PBS three times (10 min per wash) and mounted with 50% glycerol/PBS.

To label surface GluA1 on cultured neurons, neurons on coverslips were first fixed in 4% paraformaldehyde and blocked in 4% goat serum (without permeabilization) [30]. Then, the neurons were incubated with a mouse GluA1 N-terminus antibody (Millipore Sigma; 1:200) for 1 h in PBS and washed three times before being permeabilized [30]. After that, the neurons were permeabilized with 0.4% Triton X-100 and incubated with a Tubb3 antibody overnight at 4 °C for immunostaining as described above.

Fluorescence intensity was analyzed as described previously [20]. Fluorescence images were captured with a laser scanning confocal Ti microscope (Nikon, Japan). Images of brain tissues were captured using ×20 objective (numerical aperture: 1.4; working distance: 1.0 mm, corrected for 0.17 mm coverslips) with an acquisition setting of a 512 × 512 pixels resolution. Images of cultured neurons were obtained with a 60x oil immersion objective (numerical aperture: 1.4; working distance 0.13 mm, corrected for 0.17 mm coverslips) as z-series of ~6–8 images captured at 1 μm intervals with an acquisition setting of a 1024 × 1024 pixels resolution.

The quantitative analysis of immunofluorescence staining was evaluated using Image-Pro Plus 6.0 software (Media Cybernetics). Images of vezatin immunofluorescence staining in the hippocampal CA1 region were corrected for the background and segmented to isolate specific vezatin staining from nonspecific fluorescence. The hippocampal CA1 region was divided into three layers: stratum oriens, pyramidal cell layer, and stratum radiatum [13]. For each layer obtained from hippocampal CA1 region of each mouse, the MFI (calculated as integrated fluorescence intensity divided by the region of interest (ROI)) of vezatin was measured over four separate rectangular ROIs to obtain an average of MFI values. All settings and measurement parameters (immunofluorescence excitation with emission, exposure duration, background correction, and size and dimensions of ROIs) for both control and epilepsy groups were maintained at constant values throughout. A MFI value for each acquired field was then normalized to the value of the control group. The quantitative analysis of immunofluorescence staining for vezatin in human brain tissues was similar to that used for vezatin in mouse brain tissues. For cultured neurons, fluorescence images were also corrected for background and segmented to isolate the staining for specific proteins of interest from nonspecific fluorescence. The fluorescence intensity of proteins of interest was measured over three separate ROIs in each cell to obtain an average of intensity values. Intensities of GluA1 and vezatin were normalized to Tubb3 in the same ROI; the intensity of the colocalization (blue–purple) of PKA and AKAP150 was normalized to GFP in the same ROI. For each group, 12 cells were imaged and assessed per independent hippocampal neuron culture.

### Western blot analysis and coimmunoprecipitation

The western blot procedures were described previously [20, 24, 28]. We extracted total protein from mouse brain tissues, human brain tissues, and cultured hippocampal neurons using a whole protein extraction kit (Beyotime). We isolated surface protein from mouse brain tissues and cultured hippocampal neurons using the Mem-PER Plus Membrane Protein Extraction Kit (Thermo Fisher Scientific, USA) according to the instructions for transmembrane protein extraction. Next, the protein samples (50 μg per lane) were separated by SDS-PAGE on a 5% stacking gel and a 10% separating gel and then transferred to a PVDF membrane (Merck Millipore, Germany). After that, the PVDF membranes were blocked at 37 °C for 120 min in 5% skim milk and then incubated with primary antibodies overnight at 4 °C. The following primary antibodies were used: a mouse vezatin antibody (Santa Cruz Biotechnology; 1:100), mouse GAPDH antibody (Proteintech; 1:5000), rabbit GluA1 antibody (Abcam; 1:200), rabbit GluA2 antibody (Proteintech; 1:500), rabbit pGluA1-S845 antibody (Abcam; 1:1000), rabbit pGluA1-S831 antibody (Abcam; 1:1000), rabbit α-tubulin antibody (Proteintech; 1:1000), rabbit pPKA (phospho-PKA threonine 197) antibody (Cell Signaling Technology; 1:1000), rabbit PKA antibody (Cell Signaling Technology; 1:1000), mouse AKAP150 antibody (Santa Cruz Biotechnology; 1:200), mouse PP1 antibody (Santa Cruz Biotechnology; 1:200), rabbit PP2A antibody (Abcam; 1:2000), mouse PP2B antibody (Santa Cruz Biotechnology; 1:200), rabbit CaMKII-α antibody (Proteintech; 1:1000), rabbit phospho-CaMKII-α (threonine 286) (pCaMKII-α) antibody (Cell Signaling Technology; 1:1000), and mouse β-actin antibody (Proteintech; 1:5000). Then, the membranes were washed with TBST and incubated with a horseradish peroxidase-conjugated goat anti-rabbit IgG antibody (Proteintech; 1:2000) and goat anti-mouse IgG antibody (Proteintech; 1:2000) for 1 h at 37 °C. After the membranes were washed with TBST, the protein bands were visualized using enhanced chemiluminescence (ECL) reagent (Beyotime) and a Fusion FX5 image analysis system (Vilber Lourmat). The density of each band was evaluated using a Fusion FX5 image analysis system and then normalized to that of the loading control for further analysis.

For coimmunoprecipitation, the protein was extracted from mouse brain tissues using a whole protein extraction kit and then incubated with 40 μl of Protein A + G agarose beads (Beyotime) with IgG antibody (Beyotime; mouse and rabbit) for 2 h at 4 °C before centrifugation at 1000×*g* for 5 min. The supernatants were collected and incubated with mouse vezatin antibody (Santa Cruz Biotechnology), rabbit PKA antibody (Cell Signaling Technology), mouse AKAP150 antibody (Santa Cruz Biotechnology), mouse PP1 antibody (Santa Cruz Biotechnology), rabbit PP2A antibody (Abcam), mouse PP2B antibody (Santa Cruz Biotechnology), rabbit CaMKII-α antibody (Proteintech), or IgG antibody (Beyotime; mouse and rabbit) overnight at 4 °C, and then 40 μl of Protein A + G agarose beads were added and incubated for 3 h at 4 °C. The immunoprecipitates were collected and washed with lysis buffer five times after centrifugation at 1000×*g* for 5 min and then analyzed by western blotting.

### In vitro electrophysiological recordings

Whole-cell patch-clamp recordings were performed as described previously [20]. Briefly, mice were deeply anesthetized by i.p. injected sodium pentobarbital (50 mg/kg). The brains were rapidly removed from the mice, and 300-μm-thick coronal brain slices containing the hippocampus were cut with a vibratome (Leica, Germany) in ice-cold (0–4 °C) cutting solution (in mM) (2.7 KCl, 7.0 MgCl_2_, 0.5 CaCl_2_, 75.1 sucrose, 1.4 NaH_2_PO_4_, 26 NaHCO_3_, 25 glucose, and 87.3 NaCl; pH 7.35) that was continuously bubbled with carbogen (95% O_2_/5% CO_2_). Then, fresh brain slices were transferred to an incubation chamber containing artificial cerebral spinal fluid (ACSF) (in mM) (125 NaCl, 2.5 KCl, 2.0 CaCl_2_, 1.25 NaH_2_PO_4_, 25 NaHCO_3_, 10 glucose, and 1.0 MgCl_2_) and incubated at 34 °C for 60 min; the chamber was also continuously bubbled with carbogen (95% O_2_/5% CO_2_).

Hippocampal CA1 neurons were observed under an inverted phase-contrast microscope and chosen for whole-cell patch-clamp recordings. Excitatory synaptic transmission is mainly mediated by two types of ionotropic glutamate receptors: AMPARs and NMDARs [31, 32]. We, therefore, measured the AMPAR-mediated and NMDAR-mediated NST of hippocampal CA1 neurons. For mEPSC recordings, pipettes (3–8-MΩ polished glass pipettes) were filled with an internal solution (in mM) (130 CsMeSO_4_, 10 CsCl_2_, 10 HEPES, 4 NaCl, 1 MgCl_2_, 1 EGTA, 5 MgATP, 12 phosphocreatine, 5 N-methyl-d-glucamine (NMG), and 0.5 Na_3_GTP; pH 7.20). AMPAR-mEPSCs were recorded in the presence of 1 μM tetrodotoxin (TTX), 100 μM picrotoxin (PTX), and 50 μM 2-amino-5-phosphonovaleric acid (APV) at a holding potential of −70 mV in extracellular solution. NMDAR-mEPSCs were recorded in the presence of 1 μM TTX, 100 μM PTX, and 20 μM DNQX at a holding potential of −60 mV in extracellular solution. We also measured the inhibitory synaptic transmissions of hippocampal CA1 neurons: the mIPSCs of CA1 neurons were recorded in ACSF in the presence of 1 μM TTX, 20 μM DNQX, and 50 μM APV at a holding potential of −70 mV.

Next, the AMPAR-eEPSCs of hippocampal CA1 neurons were recorded. AMPAR-eEPSCs were recorded by stimulating Schaffer collaterals projecting to the CA1 region using a concentric bipolar tungsten stimulating electrode with settings of 0.05 Hz and 0.1 ms duration. The glass pipettes (3–8 MΩ) of the recording electrode were filled with the same internal solution as described above for mEPSC recordings. AMPAR-eEPSCs were recorded in the presence of 100 μM PTX and 50 μM APV in extracellular solution. The current–voltage relationship (I–V curve) of the AMPAR-eEPSCs was obtained by determining the average amplitude of ten eEPSC peaks at various holding potentials (−60 to +40 mV) [14, 33, 34]. The rectification index (RI) was calculated as the ratio of AMPAR-eEPSC amplitude at −60 mV to AMPAR-eEPSC amplitude at +40 mV (eEPSCs_-60 mV_/eEPSC_+40 mV_) [33, 34].

To record the APs of cultured hippocampal neurons in vitro, fresh hippocampal cultures were mounted on the stage and observed under an inverted phase-contrast microscope. The whole-cell patch-clamp recording was conducted to record APs at the resting membrane potential in current-clamp mode [25, 35]. The pipettes (3–5-MΩ polished glass pipettes) were filled with the following internal solution (in mM): 17.5 KCl, 0.5 EGTA, 122.5 potassium gluconate, 10 HEPES, and 4 Na_2_ATP; pH 7.2. All AP recordings were conducted with settings at I = 0 and no current was injected in AP recordings.

To monitor and acquire electrophysiological data, a MultiClamp 700B amplifier (Axon, USA) and a Digidata 1322 A interface (Axon, USA) were used. Then, the data were recorded and analyzed with pCLAMP 9.2 software (Molecular Devices, USA).

### Statistical analysis

Prior to analysis, all datasets were tested for normality and homogeneity of variance using the Kolmogorov–Smirnov test and Levene’s test, respectively. Normally distributed and homogeneous data are presented as the means ± standard errors. Comparisons between two groups were performed using unpaired Student’s two-tailed *t* test. Comparisons between multiple groups (more than two groups) considering one fixed factor were performed using one-way analysis of variance (ANOVA) followed by the Bonferroni post hoc test, and comparisons considering two fixed factors were performed using two-way ANOVA followed by the Bonferroni post hoc test. Nonnormally distributed or nonhomogeneous data are presented as the median and range, and the nonparametric Kruskal–Wallis test was used for statistical analyses. Fisher’s exact test was performed for the comparison of sex differences between patients with TLE and control subjects. Statistical significance was set at *P* < 0.05. SPSS 20.0 and GraphPad Prism 5.0 software were used for statistical analyses and to generate graphs, respectively.

## Supplementary information

Supplementary Figure Legends

Supplementary Figure S1

Supplementary Figure S2

Supplementary Figure S3

Supplementary Figure S4

Supplementary Figure S5

Supplementary Figure S6

Supplementary Figure S7

Supplementary Table S1

Supplementary Table S2

## Data Availability

The datasets generated and analyzed during this study are available from the corresponding author on reasonable request.
