# Supplementary material for: Vezatin regulates seizures by controlling AMPAR-mediated synaptic activity
Source: Cell Death Dis. 2021 Oct 12;12(10):936. doi: 10.1038/s41419-021-04233-2 (PMC8511046; doi:10.1038/s41419-021-04233-2)
Supplement: Supplementary file 1 — Supplementary Figure Legends [file 41419_2021_4233_MOESM1_ESM.docx]

**Supplementary Figure Legends**

**Figure S1.** The pattern of vezatin expression in the Mg^2+^-free medium-induced in vitro seizure-like model. (a) Representative images of Mg^2+^-free medium-induced spontaneous epileptiform discharges (SEDs) (the blue arrow indicates one SED). (b) Representative images of western blots showing vezatin expression in the control group and the in vitro seizure-like model group and (c) the corresponding statistical analysis (n = 6 independent hippocampal neuron cultures from 12 mice per group, p < 0.001). (d) Representative images of immunofluorescence staining showing that vezatin (purple) was located in neurons positive for the neuronal marker Tubb3 (green) and (e) the corresponding statistical analysis of the fluorescence intensity of vezatin in the control group and the in vitro seizure-like model group (n = 5 independent hippocampal neuron cultures from 10 mice per group; p < 0.001). Student’s t test; ***p < 0.001.

**Figure S2.** Lentiviral vectors (LVs) mediated vezatin knockdown in the mouse hippocampus. (a) Representative images of LVs (green) in the hippocampal CA1 region of mice. (b, c) Representative images of western blots showing the expression levels of vezatin weeks 2 and 4 after LV injections and the corresponding statistical analyses (n = 7 per group; week 2, Scr-seq compared with shRNA, p < 0.001; week 4, Scr-seq compared with shRNA, p < 0.001). One-way ANOVA followed by the Bonferroni post hoc test; ***p < 0.001.

**Figure S3.** LVs mediated vezatin knockdown in hippocampal neurons cultured in vitro. (a) Representative images of immunofluorescence staining showing that green fluorescent protein (GFP) (green) (expressed by the LVs) was localized in neurons positive for the neuronal marker beta-Tubulin III (Tubb3) (purple). (b) Representative images of the expression of vezatin and (c) the corresponding statistical analysis (n = 5 independent hippocampal neuron cultures from 10 mice per group; Scr-seq compared with shRNA, p < 0.001). One-way ANOVA followed by the Bonferroni post hoc test; ***p < 0.001.

**Figure S4.** The effect of vezatin knockdown on the frequency of SEDs in the Mg^2+^-free medium-induced in vitro seizure-like model. (a) Representative images of SEDs (the black arrow indicates one SED in the Scr-seq group, and the blue arrow indicates one SED in the shRNA group) and (b) the corresponding statistical analysis of the frequency of SEDs (n = 4 independent hippocampal neuron cultures from 8 mice per group; 5 cells per independent hippocampal neuron culture). Student’s t test; ***p < 0.001.

**Figure S5.** The effect of vezatin knockdown on miniature inhibitory postsynaptic currents (mIPSCs) in the hippocampal CA1 neurons of epileptic mice. (a) Representative images of mIPSCs in the Scr-seq group and shRNA group and (b) the corresponding statistical analysis of the amplitude (presented as medians and ranges; Kruskal-Wallis test) and frequency (Student’s t test) of mIPSCs in the two groups (n = 9 per group).

**Figure S6.** (a) Representative coimmunoprecipitation images demonstrating the interactions of vezatin with CaMKII-α, AKAP150, PP2B, PP2A, and PP1 in the hippocampi of mice. In the PILO-induced induced epilepsy model, (b) representative images of western blots showing the level of CaMKII-α phosphorylation, which was calculated as the ratio of phosphorylated CaMKII-α (pCaMKII-α) to total CaMKII-α, and the expression levels of PP2A and PP1 in the Scr-seq group and shRNA group, as well as (c) the corresponding statistical analyses(n = 7 per group). Student’s t test.

**Figure S7.** Representative images of immunofluorescence staining demonstrating the colocalization of PKA (blue) and vezatin (purple) in Tubb3-positive neuronal dendrites (green).
