# Supplementary figures and images for: Vezatin regulates seizures by controlling AMPAR-mediated synaptic activity
Source: Cell Death Dis. 2021 Oct 12;12(10):936. doi: 10.1038/s41419-021-04233-2 (PMC8511046; doi:10.1038/s41419-021-04233-2)

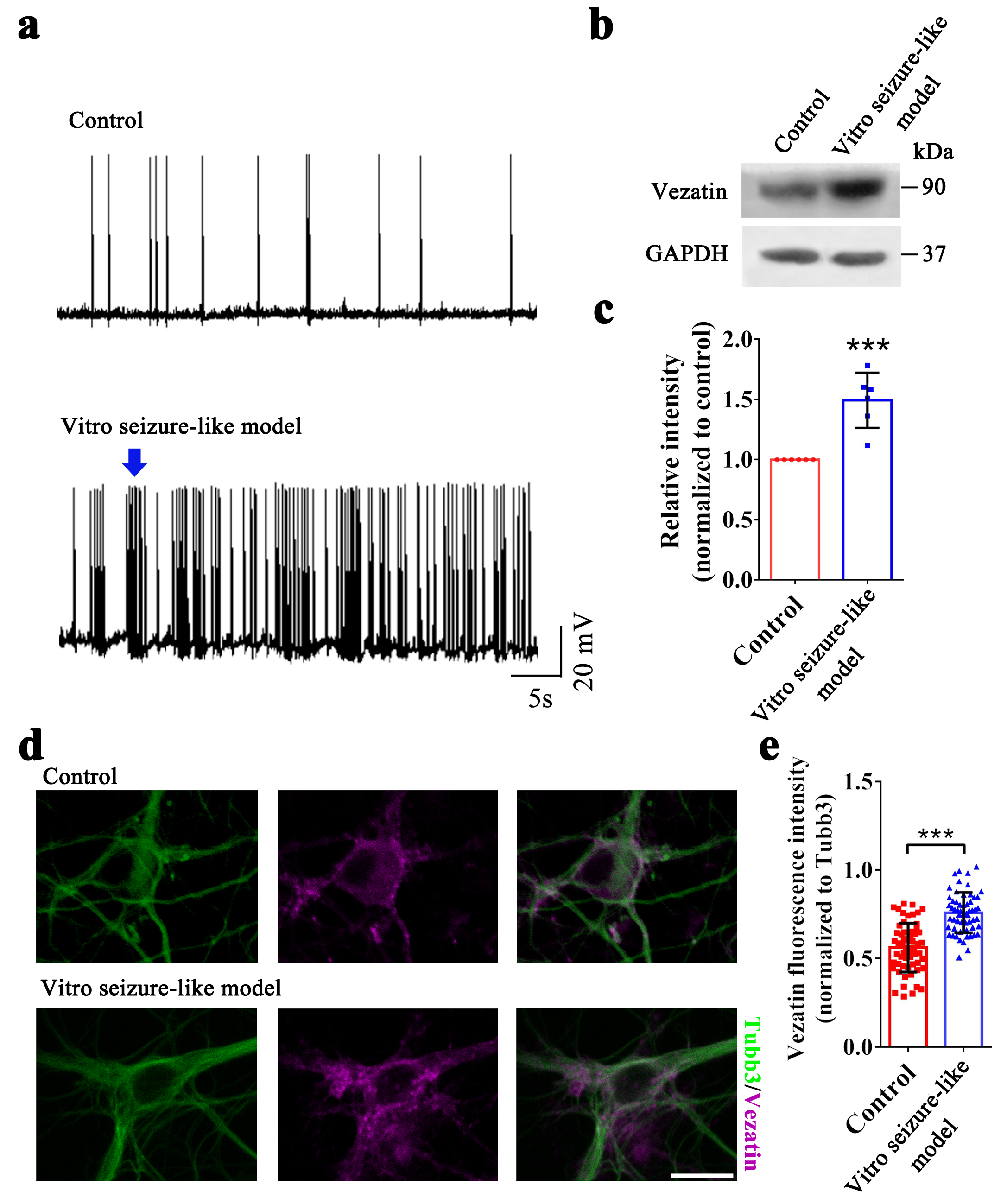

Supplement: Supplementary file 2 — Supplementary Figure S1 [file 41419_2021_4233_MOESM2_ESM.jpg]

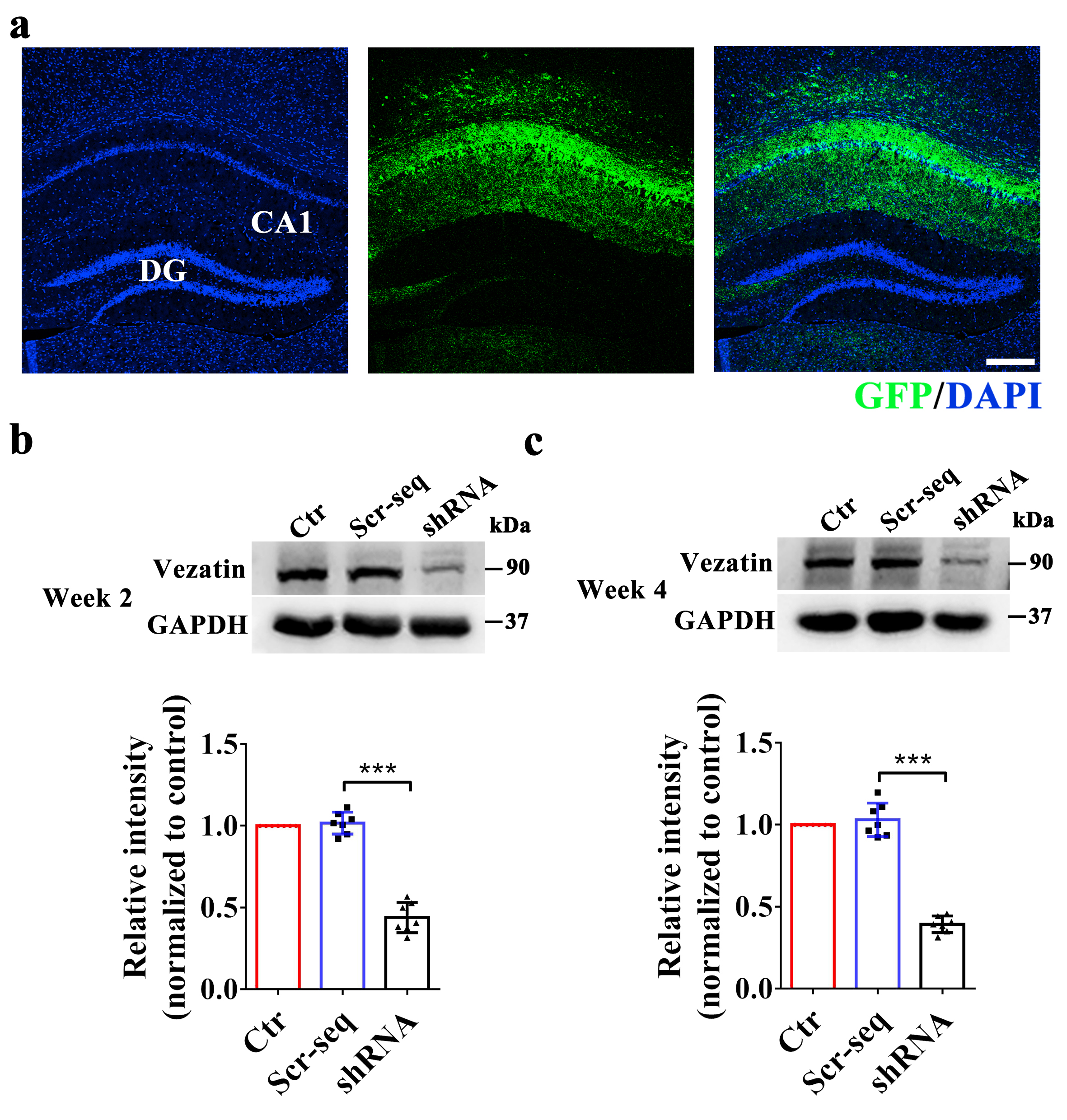

Supplement: Supplementary file 3 — Supplementary Figure S2 [file 41419_2021_4233_MOESM3_ESM.jpg]

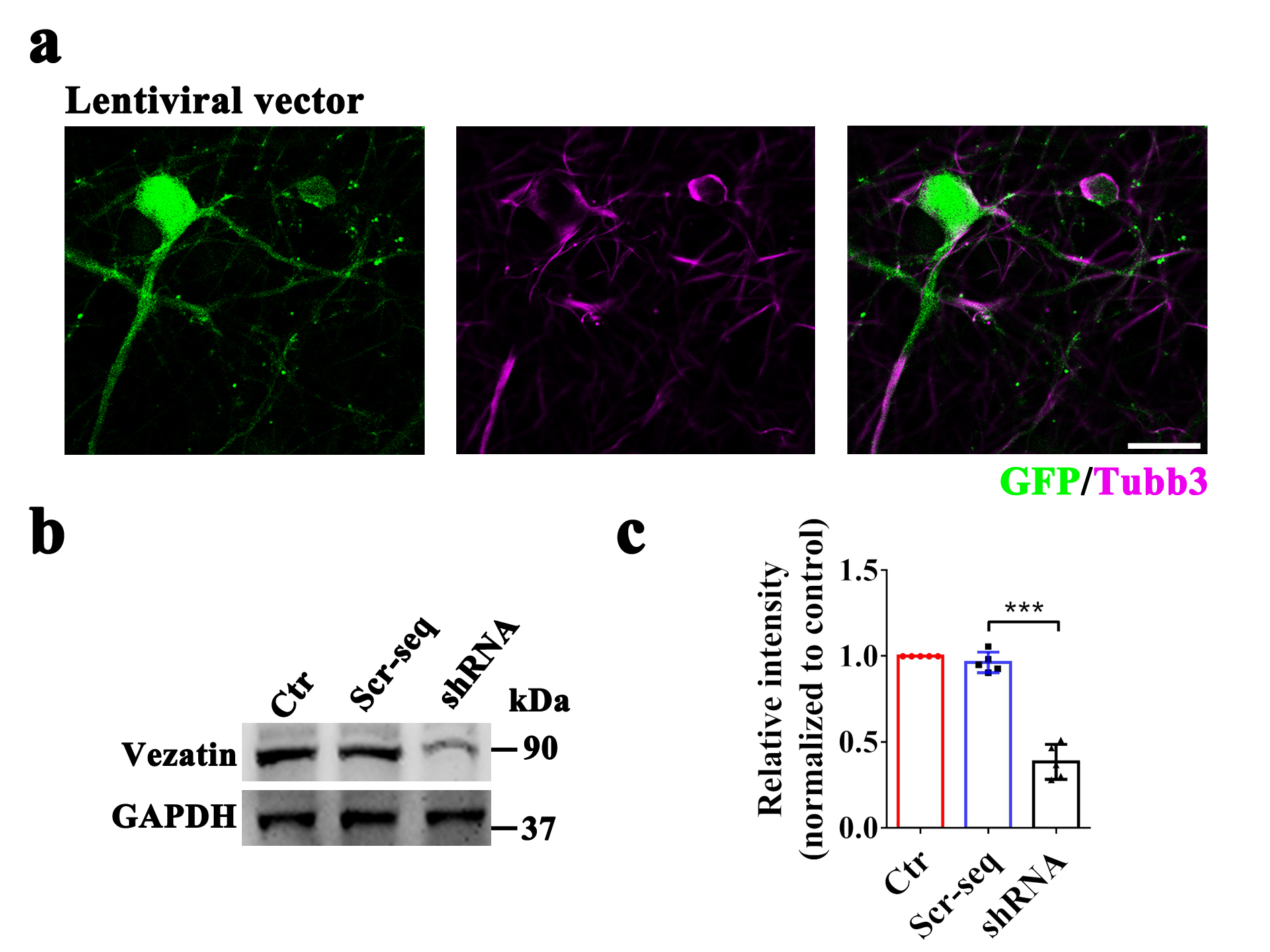

Supplement: Supplementary file 4 — Supplementary Figure S3 [file 41419_2021_4233_MOESM4_ESM.jpg]

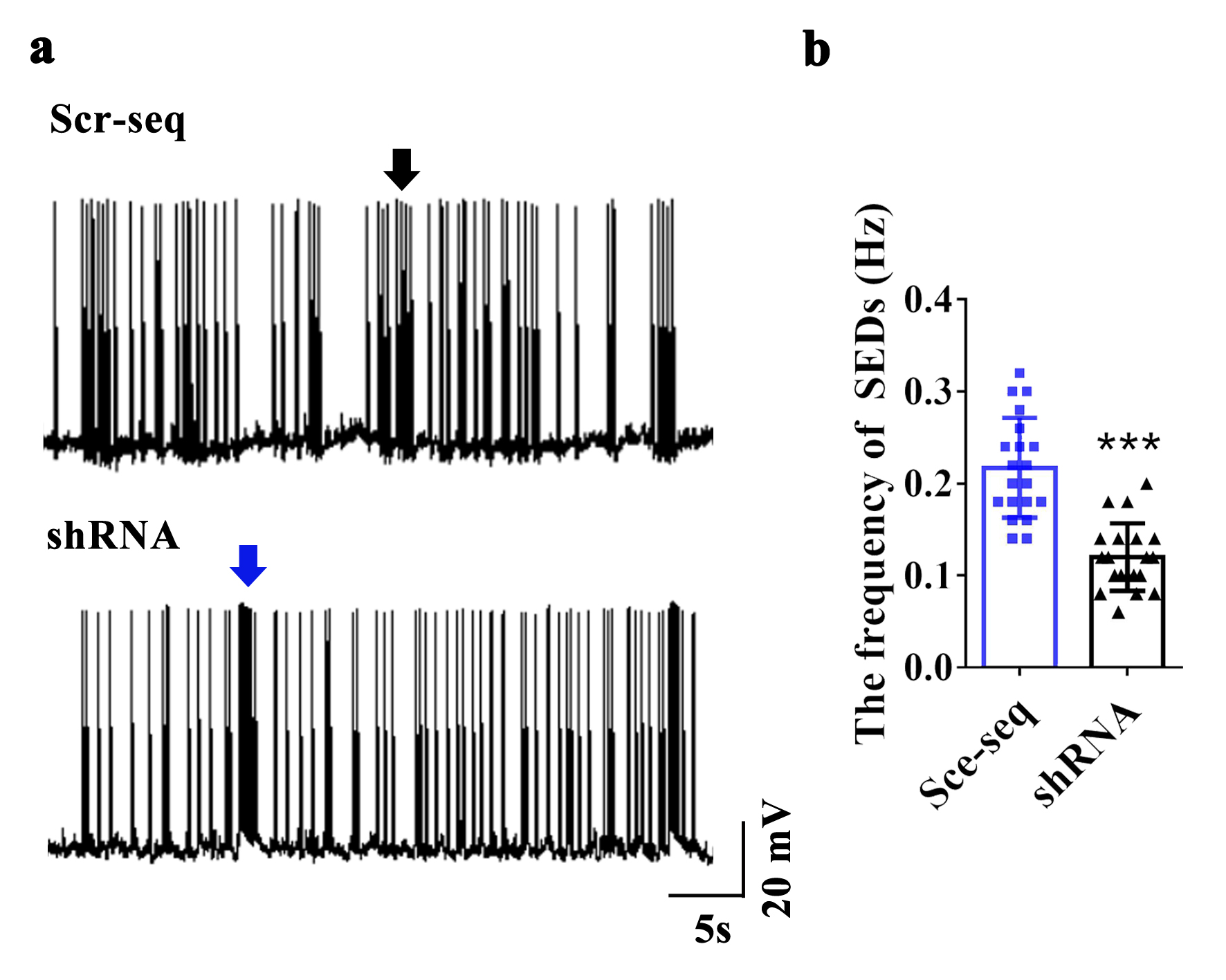

Supplement: Supplementary file 5 — Supplementary Figure S4 [file 41419_2021_4233_MOESM5_ESM.jpg]

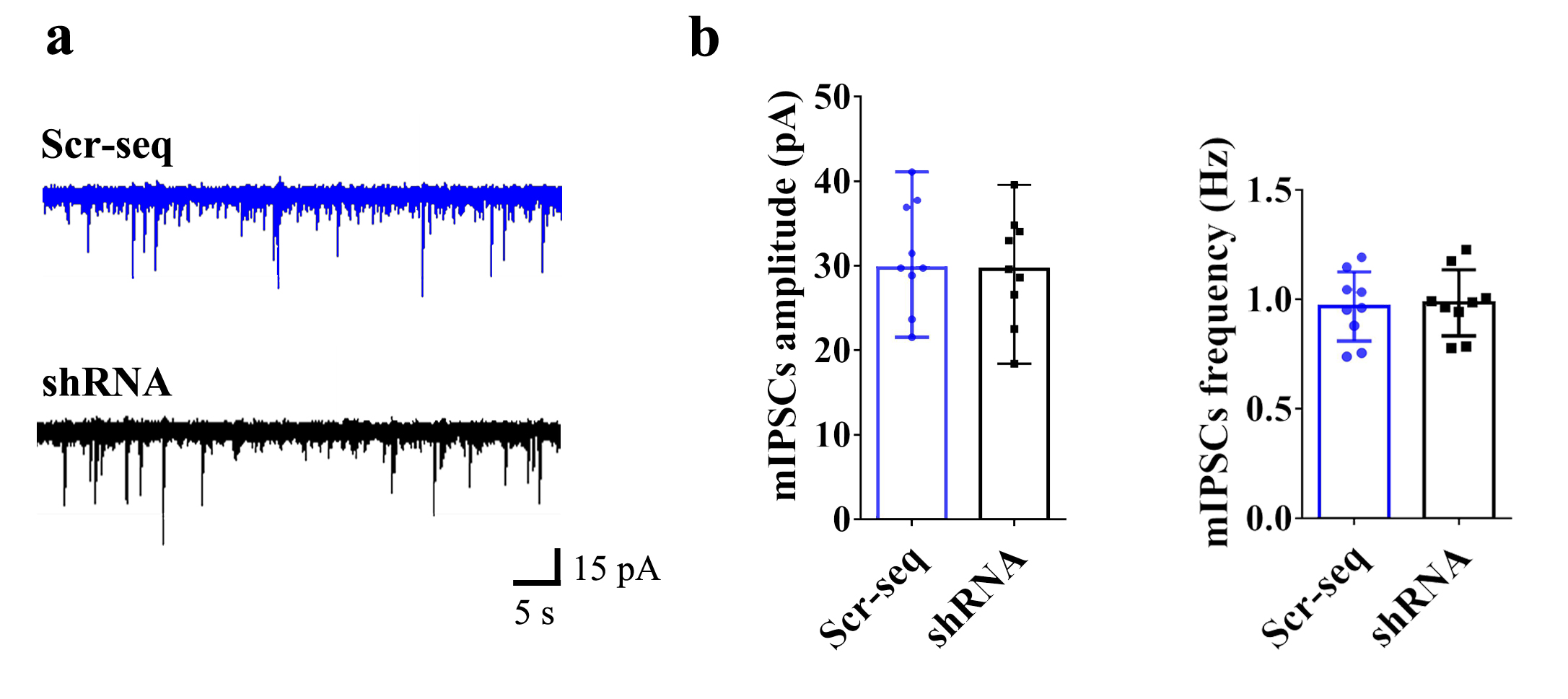

Supplement: Supplementary file 6 — Supplementary Figure S5 [file 41419_2021_4233_MOESM6_ESM.jpg]

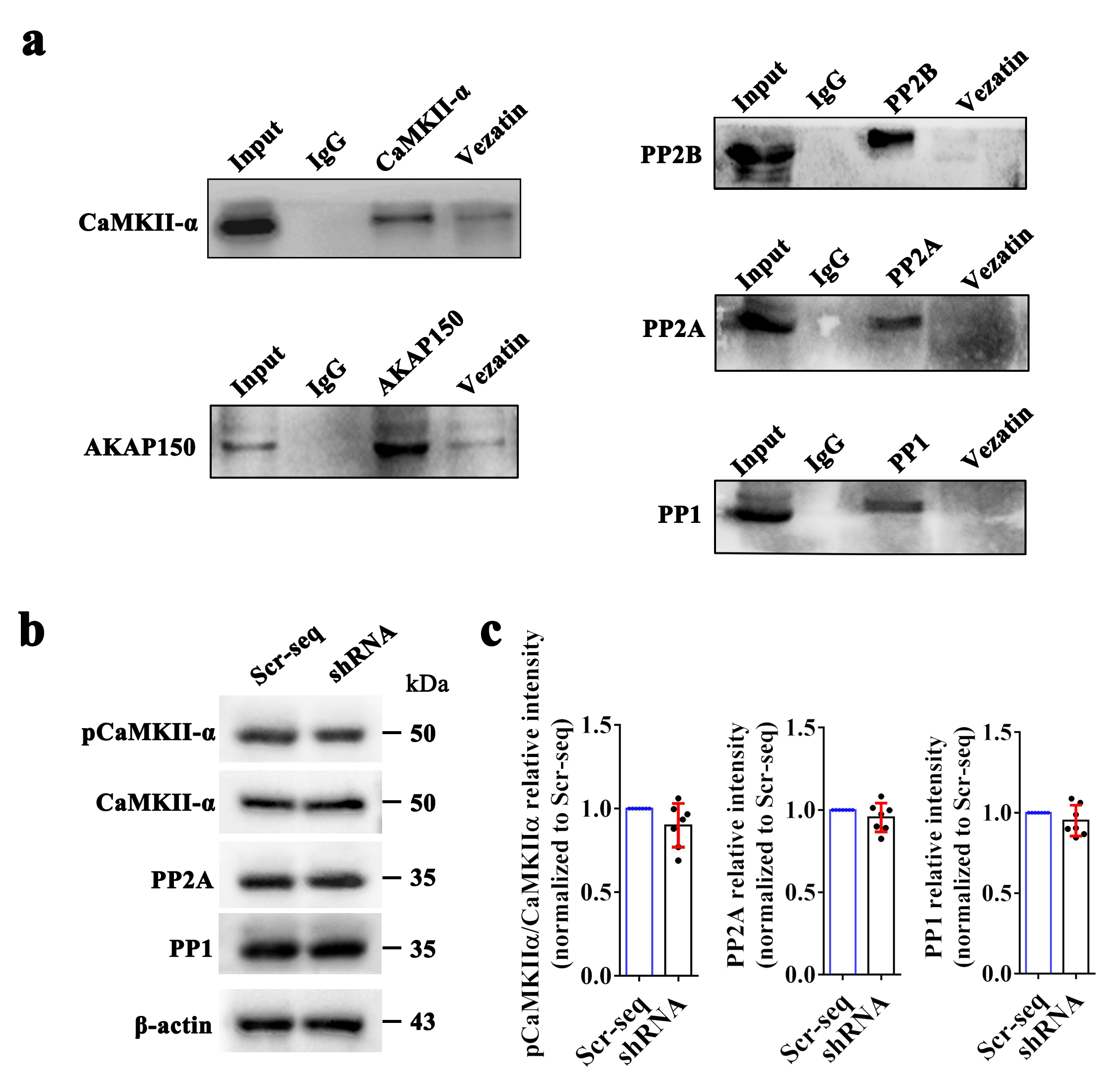

Supplement: Supplementary file 7 — Supplementary Figure S6 [file 41419_2021_4233_MOESM7_ESM.jpg]

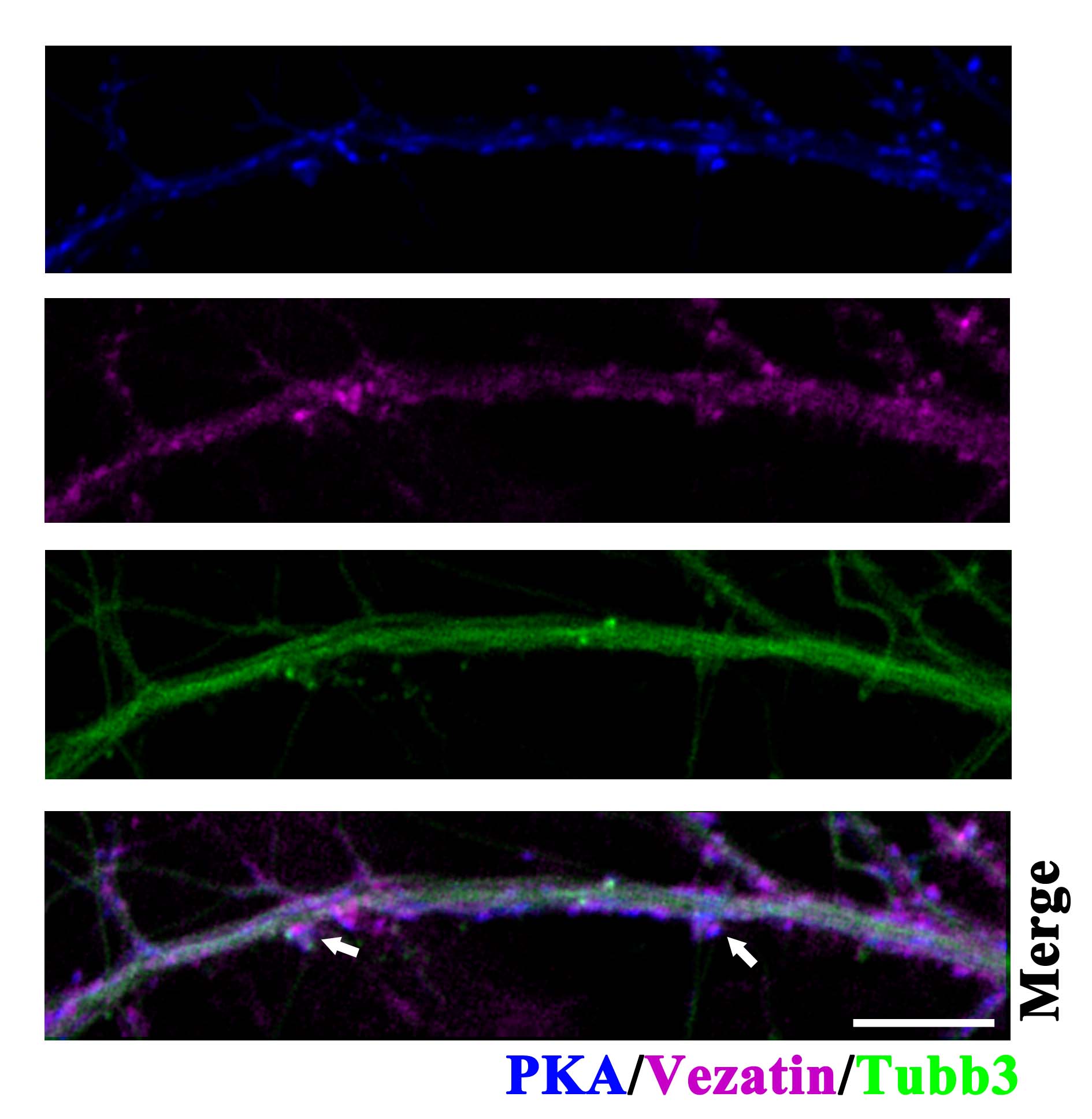

Supplement: Supplementary file 8 — Supplementary Figure S7 [file 41419_2021_4233_MOESM8_ESM.jpg]
