# Supplementary material for: Vezatin regulates seizures by controlling AMPAR-mediated synaptic activity
Source: Cell Death Dis. 2021 Oct 12;12(10):936. doi: 10.1038/s41419-021-04233-2 (PMC8511046; doi:10.1038/s41419-021-04233-2)
Supplement: Supplementary file 9 — Supplementary Table S1 [file 41419_2021_4233_MOESM9_ESM.docx]

**Supplementary Table S1**. Clinical characteristics of control patients

| No. | Sex | Age | Disease diagnosis | Resected tissue | Pathologic result |
| --- | --- | --- | --- | --- | --- |
| 1 | f | 31 | Brain trauma | RTN | Normal |
| 2 | f | 18 | Brain trauma | LTN | Normal |
| 3 | m | 49 | Brain trauma | RTN | Normal |
| 4 | f | 31 | Brain trauma | RTN | Normal |
| 5 | m | 15 | Brain trauma | LTN | Normal |
| 6 | f | 21 | Brain trauma | RTN | Normal |
| 7 | m | 28 | Brain trauma | LTN | Normal |
| 8 | f | 24 | Brain trauma | RTN | Normal |
| 9 | f | 52 | Brain trauma | RTN | Normal |
| 10 | m | 33 | Brain trauma | RTN | Normal |
| 11 | m | 17 | Brain trauma | RTN | Normal |

f: female, m: male, LTN: left temporal neocortex, RTN: right temporal neocortex.
