# Supplementary material for: Vezatin regulates seizures by controlling AMPAR-mediated synaptic activity
Source: Cell Death Dis. 2021 Oct 12;12(10):936. doi: 10.1038/s41419-021-04233-2 (PMC8511046; doi:10.1038/s41419-021-04233-2)
Supplement: Supplementary file 10 — Supplementary Table S2 [file 41419_2021_4233_MOESM10_ESM.docx]

**Supplementary Table S2**. Clinical characteristics of patients with temporal lobe epilepsy

| No. | Sex | Age at surgery (years) | Course (years) | AEDs before surgery | Resected tissue | Pathologic result |
| --- | --- | --- | --- | --- | --- | --- |
| 1 | f | 27 | 7 | CBZ, LTG, CLZ | RTN | G, NL |
| 2 | m | 31 | 8 | CBZ, LTG, PHT | RTN | G, NL, ND |
| 3 | m | 23 | 6 | CBZ, PHT, TPM | RTN | G, NL |
| 4 | m | 27 | 4 | CBZ, PHT, VPA | LTN | G, NL |
| 5 | f | 25 | 8 | OXC, VPA, TPM | RTN | G, NL, ND |
| 6 | m | 13 | 7 | OXC, PHT, VPA | RTN | G, NL, ND |
| 7 | f | 22 | 11 | OXC, VPA, TPM | LTN | G, NL, ND |
| 8 | f | 18 | 6 | OXC, VPA, LTG | LTN | G, NL |
| 9 | f | 35 | 17 | CBZ, VPA, CLZ, TPM | LTN | G, NL, ND |
| 10 | f | 14 | 7 | OXC, VPA, PHT | RTN | G, NL, ND |
| 11 | m | 21 | 12 | VPA, OXC, TPM | RTN | G, NL, ND |

f: female, m: male, AEDs: anti-epileptic drugs, CBZ: carbamazepine, VPA: valproic acid, PHT: phenytoin, CLZ: clonazepam, LTG: lamotrigine, OXC: oxcarbazepine, TPM: topiramate, LTN: left temporal neocortex, RTN: right temporal neocortex, G: gliosis, NL: neuronal loss, ND: neuronal degeneration.
